# Three-dimensional Analysis of Alveolar Bone With and Without Periodontitis

**DOI:** 10.1016/j.identj.2022.03.003

**Published:** 2022-06-25

**Authors:** Abeer A. Al-Sosowa, Mohammed N. Alhajj, Ehab A. Abdulghani, Essam Ahmed Al-Moraissi, He Zheng, Yunqing Pang, Jing Wang

**Affiliations:** a Department of Periodontics, School of Stomatology, Lanzhou University, Lanzhou, China; b Department of Periodontics, Faculty of Dentistry, Thamar University, Dhamar, Yemen; c Department of Prosthodontics, Faculty of Dentistry, Thamar University, Dhamar, Yemen; d Department of Orthodontics, School of Stomatology, Lanzhou University, Lanzhou, China; e Department of Oral and Maxillofacial Surgery, Thamar University, Dhamar, Yemen

**Keywords:** Alveolar bone density, Alveolar bone thickness, CBCT, Mandibular molars, Periodontitis

## Abstract

**Objective:**

The aim of this study was to investigate the alveolar bone density and thickness in Chinese participants with and without periodontitis.

**Methodology:**

This study was retrospective and cross-sectional in nature and used cone-beam computed tomography (CBCT) to evaluate alveolar bone loss, bone density, and bone thickness around 668 mandibular molars (344 periodontally healthy teeth and 324 teeth with periodontitis). Comparative statistical tests were done related to the age, sex, tooth type, tooth side, and degree of bone loss. The significance level was set to be *P* < .05.

**Results:**

The alveolar bone density significantly differed between the healthy and periodontitis groups (mean difference = 24.4 Hounsfield units; *P* = .007). Similarly, the alveolar bone thickness of the healthy group was significantly higher than that of the periodontitis group (4.6 ± 1.8 mm compared to 4.2 ± 1.1 mm). Teeth in females demonstrated a significantly (*P* ˂ .001) higher bone density compared with males in both healthy and compromised groups. However, males showed a significantly (*P* ˂ .05) thicker bone of the teeth than females in relation to the healthy group. The alveolar bone density and thickness in both healthy and periodontitis groups significantly differed between the first and the second molars (*P* < .001). The alveolar bone thickness had a highly significant difference (*P* < .001) between the different degrees of bone loss.

**Conclusions:**

Alveolar bone thickness and density were reduced at periodontally diseased teeth.

## Introduction

Periodontitis refers to a multifactorial chronic inflammatory disease of the periodontium, which leads to clinical attachment and alveolar bone loss. It is a major public health problem and has a plausible negative effect on general health.^1^ It causes the local and systemic release of pro-inflammatory cytokines such as interleukin-1, interleukin-6, and tumor necrosis factor-α,^2^ which are known to be linked with osteoporosis.^3^ According to the 2017 World Workshop on the Classification of Periodontal and Peri-Implant Diseases and Conditions,^4^ “Periodontitis is characterised by microbially-associated, host-mediated inflammation that results in loss of periodontal attachment.” Due to alveolar bone loss being a prominent feature of periodontal disease, bone metabolism disturbances and a decrease in the skeleton's bone mineral content, especially in the jaw, may be aggravated in the case of periodontal disease.^5^

Although clinical examination provides a lot of information required for the diagnosis of periodontitis, radiographs can give information on density and bone levels, which significantly affect diagnosis, risk assessment, and treatment outcomes. Many previous studies have verified the ability of cone-beam comouterized tomography (CBCT) and computerized tomography (CT) in measurement the grayscale value (GV) or Hounsfield units (HU) of bone tissue; therefore, they are also applicable to assessing changes in the density of the bone.^6^^,^^7^ Recently, CT and dental CBCT have predominantly been utilised to evaluate the bone density for treatment planning and placements of dental implants.^6^^,^^8^ Nonetheless, undergoing multiple CT scans during orthodontic treatment may lead to exposure of the patient to high doses of radiation. On the contrary, dental CBCT systems provide 3-dimensional images similar to CT but at a lower cost and lower dose of radiation^9^; thus, it can be utilised for continually monitoring the condition of the patient during orthodontic treatment.^10^ Later, the flat-panel detectors used in CBCT devices improved the spatial resolution, gray density range, contrast, and pixel/noise ratio.^9^ Moreover, Katsumata et al showed strong correlations of CT- and CBCT-based gray density values.^11^ Thus, we consider that CBCT is applicable for monitoring alveolar bone density changes around the teeth. However, some researchers have criticised the evaluation of bone density by using the grayscale absolute value.^12^ The scanning device, setting of image acquisition, and positioning that affects the CBCT image intensity value should be controlled to decrease CBCT-related variability in mineral density.^13^

Although the relationship between periodontitis and skeletal bone mineral density has been studied, the findings remain inconclusive.^14^^,^^15^ This lack of clarity may be due to differences between studies in the sizes of the sample, population groups, methods, and assessments of periodontal disease. Moreover, only a few publications have studied the relationship between periodontitis and local bone mineral density in the jaw, and most of them focussed on postmenopausal women.^16^

Evaluation of alveolar bone thickness of mandibular posterior teeth has been gaining attention in implantology,^17^ oral surgery,^18^ orthodontics,^19^ and endodontic surgery,^20^ which influences surgical planning^21^ and selection of the most appropriate positioning for skeletal anchorage to improve orthodontic mechanics.^19^ In addition, only 2 studies ^22^^,^^23^ were done to evaluate alveolar bone thickness with periodontitis. One of them was done in a German population by Ramanauskaite et al,^22^ who investigated the association between periodontitis and facial bone thickness. Another study, in China,^23^ evaluated alveolar bone thickness around maxillary anterior teeth with and without periodontitis.

To date, no research has studied the relationship between periodontitis and alveolar bone density and thickness around mandibular molars in a Chinese population. Therefore, this study aimed to compare alveolar bone density and thickness around mandibular molars of Chinese periodontitis patients with those of periodontally healthy individuals.

## Material and methods

### Selection of participants and sampling design

This study was a retrospective cross-sectional study approved by the ethical committee of the clinical scientific research of the Hospital of Stomatology at Lanzhou University, China (LZUKQ-2020-013).

The G*Power 3.1.9.7 software programme for Windows was used for calculation of the sample size, with a statistical power of 95%, a significance level of 5%, and an effect size of 0.57 mm estimated from the study of Al-Zahrani et al.^24^ In that study, the mean density of the posterior region in aggressive periodontitis and the healthy group was 284.2 ± 92.5 mm and 230.7 ± 95.2 mm, respectively. Thus, a total of 162 participants (81 participants in each group) would be required for the study.

The exclusion criteria included (1) patients with systemic diseases, pregnant persons, smokers, or those who received medications that influence bone metabolism; (2) patients with missing molar teeth (except for third molars); (3) patients with local conditions that influence bone quality (eg, fenestration, cysts, tumors, surgical history, trauma, or prior orthodontics); and (4) those with teeth with periodontal treatment in the previous 6 months,^25^ root canal therapy, or restorative therapy (eg, Class II and Class V restoration that extends subgingivally and may later affect the periodontium).

Participants were grouped into 2 age categories (≤30 years, >30 years). The periodontitis group was already diagnosed clinically and through CBCT, according to the 2017 classification system of periodontal diseases,^26^ by a specialist in periodontology with more than 15 years of experience at the Hospital of Stomatology in Lanzhou University, China. In our study, we classified the periodontitis group according to the severity of alveolar bone loss into the following groups^27^: (a) no bone loss: in the radiographic image, the distance from the cement-enamel junction (CEJ) to the crest of alveolar bone was 1 to 2 mm; (b) mild: radiographic bone loss of less than one-third of the root length; (c) moderate: radiographic bone loss of one-third to one-half of the root length; and (d) severe: radiographic bone loss of one-half or more of the root length in random sites.

### Radiographic acquisition

All participants had undergone a 3D scan using an i-CAT® imaging device (Imaging Sciences International) with the field of view (FOV) of 16.0 × 13 cm and tube voltage of 120 kV for a total scan time of 8.9 seconds, with the voxel size at 0.3 mm. The data were stored in DICOM format (Digital Imaging and Communications in Medicine). Invivo 5.2 software programme (Anatomage) was used to analyse the raw data obtained from the CBCTs.

### Three-dimensional measurements

**Fig 1:**
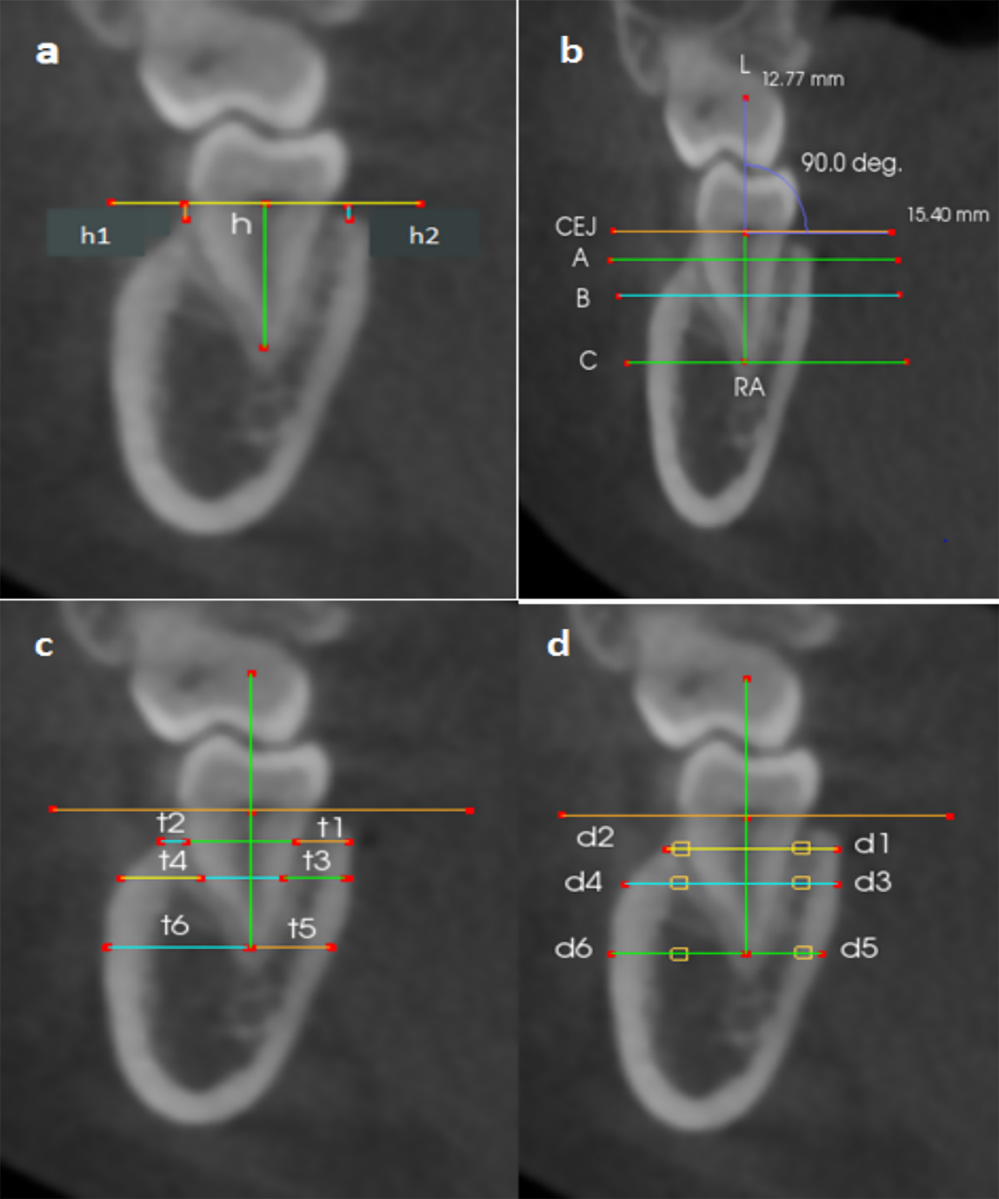
Diagrams of thickness and density of the alveolar bone measurements. A, Root length (h) was marked by bisecting the pulp chamber from the midpoint of the cement-enamel junction (CEJ) line to the root apex (RA). The bone loss was marked by a vertical distance to the crest of alveolar bone from the CEJ line buccally and lingually (h1 and h2, respectively). B, Line A, line B, and line C were drawn perpendicular to the long axis of the teeth L. C, Lingual and buccal bone thicknesses were measured at 1 mm apical to the alveolar crest (t1, t2), mid-root level (t3, t4), and apical level (t5, t6). D, Lingual and buccal bone densities were obtained at 1 mm apical to the alveolar crest (d1, d2), mid-root level (d3, d4), and apical level (d5, d6). The values were registered in the area size of 1 mm at a distance of 3.5 mm from the root.

Each CBCT image in coronal slices was extracted to evaluate the alveolar bone density and thickness of mandibular molars. Measurements involved the degree of bone loss, alveolar bone density, and thickness. The alveolar bone loss severity was estimated by the bone loss percentage and was calculated by [(h1 or h2 − 2 mm)/(h − 2 mm)] × 100%,^23^ where h1 and h2 represented the buccal and lingual vertical distance from the CEJ line to the crest of alveolar bone. h represented the root length (ie, distance from the intermediate point of the CEJ to the apex of the root [RA]) (Figure 1A). Three reference lines were drawn perpendicular to the long axis of each tooth. Line A was drawn at 1 mm from the alveolar crest apically; line B was drawn at the intermediate point between the CEJ and the root apex line, and line C was drawn at the root apex (Figure 1B). The alveolar bone thickness was measured in mm, as shown in Figure 1C. After we did several trials of measuring, we found that the distance of 3.5 mm from the midline buccally and lingually was the most appropriate point undertaken to limit the measurement of bone density to the bone. The size of the measured area was adjusted to be equal in all cases, and it was 1 mm^2^, as shown in Figure 1D.

The average of the 3 levels of each buccal and lingual measurement was calculated, and the differences in alveolar bone density and thickness between healthy and periodontitis groups were analysed according to the following parameters: age, sex, tooth type, tooth side, and severity of the bone loss.

### Statistical analysis

SPSS (Statistical Package for Social Sciences) version 22.0 (IBM) was used for all statistical data analysis. The test of normality was utilised using the Shapiro–Wilk test. Accordingly, the non-parametric Kruskal–Wallis test and Wilcoxon Mann–Whitney U tests were utilised to compare the studied groups. Spearman correlation coefficient was used to predict the correlation analysis. A multivariate analysis was performed to explore the interaction effect on the combined dependent variables for each case group separately (healthy and periodontitis). A *P* value <.05 was set to be statistically significant.

For reliability of the measurements, a random selection of 20% of the selected sample (30 CBCTs) was remeasured within a 2-week interval in between by the same observer and by another examiner. The intraclass correlation coefficient (ICC) was used to measure the intra- and interobserver agreements.

## Results

CBCT radiographs of 167 participants, including 81 diagnosed with periodontitis and 86 periodontally healthy individuals, were evaluated in this study. The mean ages of the periodontally compromised and healthy groups were 29.5 ± 7.0 and 25.5 ± 5.2 years, respectively. There were 96 (57.5%) females and 71 (42.5%) males with a mean age of 26.5 ± 6.0 and 28.8 ± 6.8 years, respectively. The study investigated a total of 668 teeth, including 344 periodontally healthy and 324 periodontally compromised teeth. More details are shown in Table 1.

**Table 1:** Characteristics of the study sample.

	All teeth	Healthy teeth	Periodontally compromised teeth
	n = 668	n = 344	n = 324
Age group, y			
≤30	500 (74.9)	300 (87.2)	200 (61.7)
>30	168 (25.1)	44 (12.8)	124 (38.3)
Sex			
Male	284 (42.5)	152 (44.2)	132 (40.7)
Female	384 (57.5)	192 (55.8)	192 (59.3)
Tooth type			
First molars	334 (50.0)	172 (50.0)	162 (50.0)
Second molars	334 (50.0)	172 (50.0)	162 (50.0)
Tooth side			
Right	334 (50.0)	172 (50.0)	162 (50.0)
Left	334 (50.0)	172 (50.0)	162 (50.0)
Severity of bone loss			
Healthyperio*	102 (15.3)	NA	102 (31.5)
Mild	50 (7.5)	NA	50 (15.4)
Moderate	47 (7.0)	NA	47 (14.5)
Severe	125 (18.7)	NA	125 (38.6)

Data are No. (%).

⁎ Healthyperio refers to healthy teeth in periodontally compromised patients. NA, not applicable.

The result of the ICC test revealed a high consistency, ranging from 0.951 to 0.999 for intra-examiner and from 0.855 to 0.994 for inter-examiner reliability analysis, with a *P* value <.001.

As shown in Table 2, the overall bone density of the healthy group was significantly higher than that of the periodontally compromised group (difference = 24.4 HU; *P* = .007). Similarly, a highly significant difference (*P* < .001) was found between healthy and periodontally compromised groups in relation to the overall bone thickness, with a higher value in favour of the healthy group (4.6 ± 1.8 mm compared to 4.2 ± 1.1 mm).

**Table 2:** Differences in alveolar bone density (HU) and thickness (mm) amongst the study groups.

Differences based on the diagnosis					
		Healthy	Periodontitis	Difference (95% CI)	*P* value*
Density		800.0 ± 133.1	775.6 ± 141.6	24.4 (3.5 to 45.2)	.007
Thickness		4.6 ± 1.8	4.2 ± 1.1	0.5 (0.2 to 0.7)	˂.001
Differences based on age					
		≤30	>30	Difference (95% CI)	*P* value*
Healthy	Density	804.9 ± 134.8	766.2 ± 116.7	38.7 (−3.4 to 80.9)	.175
	Thickness	4.6 ± 2.0	4.7 ± 0.6	0.0 (−0.6 to 0.6)	.198
Periodontitis	Density	786.6 ± 149.9	757.9 ± 125.5	28.7 (−3.0 to 60.5)	.068
	Thickness	4.2 ± 1.1	4.1 ± 1.1	0.0 (−0.2 to 0.3)	.876
Differences based on sex					
		Male	Female	Difference (95% CI)	*P* value*
Healthy	Density	753.7 ± 113.2	836.6 ± 136.4	−83.0 (−109.5 to −56.5)	˂.001
	Thickness	4.7 ± 0.7	4.6 ± 2.4	0.0 (−0.4 to 0.4)	.017
Periodontitis	Density	737.7 ± 122.1	801.7 ± 148.3	−64.0 (−93.7 to −34.3)	˂.001
	Thickness	4.3 ± 1.1	4.1 ± 1.1	0.3 (0.0 to 0.5)	.072
Differences based on tooth type					
		First molar	Second molar	Difference (95% CI)	*P* value*
Healthy	Density	871.1 ± 122.9	728.9 ± 101.3	142.2 (118.3 to 166.1)	˂.001
	Thickness	4.1 ± 0.5	5.2 ± 2.4	−1.1 (−1.4 to −0.7)	˂.001
Periodontitis	Density	817.9 ± 153.8	733.4 ± 113.8	84.5 (54.9 to 114.1)	˂.001
	Thickness	3.6 ± 1.0	4.7 ± 1.0	−1.1 (−1.3 to −0.9)	˂.001
Differences based on tooth side					
		Right	Left	Difference (95% CI)	*P* value*
Healthy	Density	787.8 ± 131.5	812.1 ± 133.9	−24.3 (−52.5 to 3.8)	.123
	Thickness	4.6 ± 0.7	4.7 ± 2.5	−0.2 (−0.5 to 0.2)	.725
Periodontitis	Density	774.6 ± 143.2	776 ± 140.3	−2.1 (−33.0 to 28.9)	.927
	Thickness	4.2 ± 1.1	4.1 ± 1.1	0.1 (−0.1 to 0.4)	.372

*Mann–Whitney U test was used.

⁎ *P* value is considered significant at <.05. HU, Hounsfield units.

Females demonstrated a significantly (*P* < .001) higher bone density of the teeth compared with males in both healthy and periodontally compromised groups. However, in the healthy group only, teeth in males showed a significantly (*P* < .05) thicker bone than in females (Table 2).

The alveolar bone density of the first molars was significantly (*P* < .001) higher than that of the second molar in both healthy and periodontitis groups. However, the alveolar bone was thicker in the second molar than in the first molar in both healthy and periodontally compromised groups, with a highly significant difference (*P* < .001, Table 2).

The multivariate analysis revealed no significant interaction effect amongst age, sex, tooth type, and tooth side on the combined dependent variables (healthy group: Wilks’ λ = 1.000; *P* = .965; periodontitis group: Wilks’ λ = 0.998; *P* = .771).

There was a highly statistically significant difference (*P* < .001) between the different types of bone loss in relation to alveolar bone thickness (Table 3). The pairwise comparison, based on Bonferroni correction, showed that the only significant differences were between healthyperio and mild groups and between healthyperio and severe groups (*P* = .036 and *P* = .001, respectively).

**Table 3:** Differences in bone density (HU) and thickness (mm) based on the severity of bone loss.

	Density	Thickness
Healthyperio*	786.7 ± 126.1	4.5 ± 0.8
Mild	763.5 ± 125.9	4.1 ± 1.5
Moderate	763.0 ± 211.4	4.0 ± 1.0
Severe	762.8 ± 126.0	3.9 ± 1.1
*P* value†	.821	˂.001

⁎ Healthyperio refers to healthy teeth in periodontally compromised patients.

† Kruskal−Wallis test was used. *P* value is considered significant at <.05. HU, Hounsfield units.

The results of the correlational analysis, as seen in Table 4, demonstrated that the density had a negative correlation with the thickness in both healthy and periodontitis groups.

**Table 4:** Correlation between alveolar bone density (HU) and thickness (mm).

	Density	Thickness	*P* value*
Healthy	1.000	−0.591**	<.001
Periodontitis	1.000	−0.269**	<.001

⁎ Spearman correlation coefficient test.

⁎⁎ *P* value is considered significant at <.01. HU, Hounsfield units.

## Discussion

Our study utilised CBCT to assess alveolar bone density and thicknesses of the mandibular molars in patients with periodontitis and healthy individuals.

Some studies have evaluated mandibular bone mineral density and periodontitis.^14^^,^^24^^,^^25^^,^^28^^,^^29^ Klemetti et al,^28^ using dental radiographs, reported higher bone density in the regions affected by periodontitis. However, similar to our results, Von et al,^29^ using dual-energy X-ray absorptiometry, found that severe periodontitis in young adults seems to be a local factor associated with relatively low bone mineral content in the jaws without systemic alterations of bone mineral content, bone mineral density, and bone metabolism. Takaishi et al^14^ suggested a significant negative association between mandibular bone mineral density and levels of periodontal attachment. Similarly, Öztürk et al,^25^ using dual-energy X-ray absorptiometry, found that the mandibular bone mineral density of the periodontally healthy individuals was higher than that in the periodontitis group. Al-Zahrani et al,^24^ using CBCT in a Saudi Arabian population, found that alveolar bone density was different between the individuals with and without aggressive periodontitis; however, their result was not statistically significant.

Our result found that alveolar bone density was not different between the analysed teeth of the different age groups, which is not in line with the findings of Öztürk et al^25^ and Horner and Devlin,^30^ who found that mandibular bone mineral density decrease with age. This disagreement might be due to the differences in ethnic groups, sample size, imaging technique, and methods of measurement.

The findings of the current study showed that the females had a higher bone density compared with males, which may be related to the differences in metabolic and calcium intake; this finding is not similar to that of Öztürk et al^25^ and Pluskiewicz et al.^31^ The inconsistency between the findings may be due to differences in areas of bone that had been measured, methods of measurement, and the type of radiographic scans.

Related to the alveolar bone thickness, the results of the recent research found that there was a significant reduction in the alveolar bone thickness of the patients with periodontitis compared to healthy individuals, which may challenge implant therapy. This result is similar to that of Ramanauskaite et al,^32^ who assessed the effect of local pathologies, including periodontitis, on the dimensions of facial alveolar bone at tooth sites and concluded that the thickness of facial bone was reduced at periodontitis sites.

In agreement with the current study, Kim et al^33^ found that the mandibular buccal and lingual cortical bone was thicker in men than in women. Similarly, Zhang et al^23^ and Wenjian et al^34^ explored the alveolar bone thickness at the anterior maxillary teeth by using CBCT and reported that the alveolar bone thickness was lower in females than in males. This can be related to the differences in masticatory forces and skeletal growth. On the other hand, Hessam et al^35^ reported no significant differences in bone thickness related to sex.

The result of the current study showed that alveolar bone thickness had no significant differences related to the age groups, which is consistent with that of Hessam et al.^35^

This study analysed the bone thickness in lower first and second molar teeth and found that the bone thickness at the second molars was greater than that of the first molars, which is in line with the previous studies in Iranian,^36^ American,^37^ and Swiss populations.^38^ This result may be related to the location of the external oblique ridge in the buccal region of the second molar, causing the bone thickness to be increased.

According to the current findings, the alveolar bone thickness differed with different types of bone loss, which is in accordance with the result found by Zhang et al,^23^ who reported that when the loss of alveolar bone was one-half of the root length, the thickness of the residual buccal bone significantly increased, whereas when the bone loss was one-third of the root length, the thickness of the residual palatal bone decreased.

Al-Masri et al^39^ used CBCT to evaluate the relationship between the density and thickness in orthodontically untreated adults with lower incisors and found a negative correlation between the apical thickness and density of both buccal and lingual surfaces, which is similar to our results. This negative correlation may be attributed to the compensatory mineralisation of the alveolar bone as a defence mechanism in the trabecular portion of the jaw against bone loss.^40^

According to the As Low As Reasonably Achievable principle, the radiographic examination's ideal goal is to achieve as much information as possible about the jawbone whilst minimising the costs and the radiation burden to the patient. CBCT is a relatively recent imaging method that produces 3-dimensional images similar to CT but at a lower cost and radiation dose.^41^ Several studies verified a significant correlation between CT and CBCT in bone density measurement.^42^^,^^43^ However, the use of CBCT GV in bone density evaluation was not recommended by some researchers when used as an absolute value.^12^^,^^44^ In the current study, standardised CBCT image voxel values were compared between healthy and periodontitis groups, which eliminated the need for having absolute values.^45^ Furthermore, Valiyaparambil et al^45^ estimated the association between HU and dental CBCT GVs. They suggested that although the CBCT GVs (voxel values) were not absolute, the CBCT dimensional accuracy was similar to CT.

Although the periodontitis group in our study was diagnosed previously by a specialist, the clinical parameters were not included for comparison in the analysis, which could be considered a limitation of the study. Another limitation in this study includes the use of CBCT to assess the alveolar bone density and thickness only in mandibular molars. In addition, the apical position of the bone level measured by CBCT scans did not reflect the periodontal condition of the participant but the anatomic position of the alveolar bone in the mandibular molar area. Hence, further studies are recommended to evaluate the differences in alveolar bone density and thickness around anterior and posterior teeth in the upper and lower jaw with and without periodontitis utilising CBCT, CT, and dual-energy X-ray absorptiometry.

## Conclusions

Considering the limits of our study, it can be concluded that alveolar bone density and thickness of the mandibular molars differed between healthy individuals and patients with periodontitis. Furthermore, there was a negative correlation between alveolar bone density and thickness in both healthy and periodontitis groups.

## Ethics approval

The study was approved by the Ethical Committee of the Hospital of Stomatology at Lanzhou University, China (LZUKQ-2020-013).

## Author contributions

Abeer A. Al-Sosowa: Conceptualisation, data curation, formal analysis, investigation, methodology, project administration, resources, software, supervision, validation, visualisation, writing – review and editing. Mohammed N. Alhajj: Conceptualisation, formal analysis, investigation, writing – original draft. Ehab A. Abdulghani: Conceptualisation, data curation, writing – original draft. Essam Ahmed Al-Moraissi: Investigation, writing – review and editing. He Zheng: Data curation, writing – original draft. Yunqing Pang: Conceptualisation, data curation, methodology. Jing Wang: Conceptualisation, supervision, visualisation, writing – review and editing.

## Conflict of interest

None disclosed.
